# Neck Injury Comorbidity in Concussion-Related Emergency Department Visits: A Population-Based Study of Sex Differences Across the Life Span

**DOI:** 10.1089/jwh.2018.7282

**Published:** 2019-04-22

**Authors:** Mitchell Sutton, Vincy Chan, Michael Escobar, Tatyana Mollayeva, Zheng Hu, Angela Colantonio

**Affiliations:** ^1^ Toronto Rehabilitation Institute–University Health Network, Toronto, Canada.; ^2^ Dalla Lana School of Public Health, University of Toronto, Toronto, Canada.; ^3^ Department of Occupational Sciences and Occupational Therapy, University of Toronto, Toronto, Canada.; ^4^ Rehabilitation Sciences Institute, University of Toronto, Toronto, Canada.; ^5^ Institute for Clinical and Evaluative Sciences, Toronto, Canada.

**Keywords:** concussion, neck injury, sex, motor vehicle, sports

## Abstract

***Background:*** The cervical spine region can be especially vulnerable to concurrent injury in concussion, with research suggesting that females may be at greater risk due to their weaker and anatomically distinct necks. The main objective of our research was to study sex differences in the rate of neck injury comorbidity across the life span among patients with a concussion diagnosis in the emergency department (ED) setting, by cause of injury (motor vehicle collisions [MVC] and sports).

***Materials and Methods:*** All patients with a first concussion-related ED visit between fiscal years 2002/2003 and 2011/2012 (inclusive) in Ontario were identified in population-based health administrative data using the International Statistical Classification of Diseases and Related Health Problems, Tenth Revision, Canada (ICD-10-CA) codes. Age-dependent odds ratios of comorbid neck injury for sex were estimated using polynomial multivariable logistic regression models, adjusting for sociodemographic characteristics.

***Results:*** Females with a concussion had significantly higher odds of sustaining a comorbid neck injury between the ages of 5–49 years for all concussion-related ED visits, 15–49 years for MVC-related concussion ED visits, and 10–39 years for sports-related concussion ED visits, holding all other covariates in the model constant.

***Conclusions:*** These results support the consideration of increased screening for comorbid neck injuries, particularly for females, to allow for early intervention. Furthermore, the increased risk of comorbid neck injury in females with a concussion-related ED visit was age-dependent, with the interaction between sex and age following a nonlinear trend. As such, future studies on concussions should consider linear and nonlinear sex and age interactions.

## Introduction

Traumatic brain injury (TBI) remains a major cause of death and disability in the United States,^1^ with ∼3.5 million new injuries occurring each year^2^ with an annual estimated economic cost of more than $70 billion (including both indirect and direct medical costs).^3^ The majority of injuries (75%) are mild in severity^2^ and are frequently referred to as concussions.^4^ The overall incidence of TBI is higher among males^2^; however, clinical studies have shown that females are at a higher risk of concussions in sports with similar rules.^5–9^ Furthermore, length to recovery post-injury has been found to be longer among females, who are also more likely to have persistent symptoms for more than a month.^10,11^ To date, although there has been a plethora of research on TBI and concussion, there is a paucity of studies assessing the prevalence of comorbid neck injuries among males and females with concussion, across the life span and by cause of injury.

The important role of a co-occurring (comorbid) neck injury (*i.e.*, injury to the cervical spine region) with concussion is reflected in the latest consensus statement on sports-related concussions.^4^ Literature supports that whiplash-associated disorders and concussions share similar clinical presentations^12–16^ and have been found to commonly co-occur.^17^ In addition, injury to the cervical spine region is particularly vulnerable to concurrent concussion due to its proximity to the head and the lack of protection relative to other regions (*i.e.*, thoracic and lumbosacral) of the spine.^18^ Evidence also supports that females have weaker and anatomically distinct necks than males,^19,20^ which suggests that the relationship between neck injuries and concussions may be sex-dependent and that biological differences between males and females may be responsible for the observed increased vulnerability among females to sustain whiplash in motor vehicle collisions (MVC),^21,22^ poorer prognosis following injury,^23,24^ and increased risk of concussion.^25^

While previous studies on neck injuries and TBI have incorporated sex as a risk factor,^26,27^ they were based on samples with mixed injury severity from major trauma centers and were not population-based. To expand our understanding of neck injury comorbidity at a population level among patients with TBI that are mild in injury severity, a population-based study of all patients with their first concussion-related emergency department (ED) visit in a publicly funded health care system was conducted. Due to the proclivity of concussions and neck injuries in sports and MVC,^5–9,21,22^ this study focused on three distinct populations in the ED: sports-related concussions, MVC-related concussions, and all concussions (regardless of cause of injury). It was hypothesized that the rate of comorbid neck injury among patients in the ED with a concussion diagnosis would be higher among female patients than among male patients, and the magnitude of risk would be age-dependent, controlling for injury-related factors (mechanism, intention of injury [against the injured person]) and other sociodemographic factors (income, rurality of residence).^28^

## Materials and Methods

The Research Ethics Board at the Toronto Rehabilitation Institute-University Health Network (TRI-UHN) and the University of Toronto Research Ethics Board approved this study.

### Data source

Data on ED visits in Ontario, which has a universal health care system that covers all publicly funded health care services, were extracted from the Canadian Institute for Health Information (CIHI) National Ambulatory Care Reporting System (NACRS).^29^ These data were obtained through the Ontario Cancer Data Linkage Program (cd-link). The cd-link is an initiative of the Ontario Institute for Cancer Research/Cancer Care Ontario Health Services Research Program whereby risk reduced coded data from the Institute for Clinical Evaluative Sciences are provided directly to researchers with the protection of a comprehensive data use agreement.

### Sample

Patients with a first concussion-related ED visit between fiscal years 2002/2003 and 2011/2012 (inclusive) were identified using the International Statistical Classification of Diseases and Related Health Problems, Tenth Revision, Canada (ICD-10-CA) code S06.0 in any of the 10 diagnosis fields in the NACRS.^30^ To maintain sample homogeneity in terms of injury severity, only patients with mild injury severity, as defined by the Abbreviated Injury Score of less than three,^31,32^ were included.

### Variables

Sociodemographic variables included age, sex, income, rurality, and fiscal year of ED discharge. Income and rurality were examined, as previous research suggests that persons of lower socioeconomic status and rural residence have higher rates of ED visits.^33–35^ Fiscal year of ED discharge was also examined to account for the increased awareness of concussion-/brain-related injuries in recent years.^36–38^ Age was categorized into 5-year age groups, ranging from age 0 to 4 years to 85+ years and treated as an ordinal variable in the logistic models (described below). Sex was categorized as male and female and was the main variable of interest in these models. Income was assessed using five income quintiles, with one representing the lowest and five the highest level of income, adjusted for household size and community.^39^ Rural residence was defined based on the individual's postal code, using the Canadian Postal Code designation for rural.^40^

Injury-related variables included mechanism of injury and intention of injury, which were defined as per the Centers for Disease Control and Prevention Injury Matrix.^41^ Mechanism of injury was categorized into falls, MVC, stuck by/against, other transportation (*e.g.*, pedal cyclist), and other. Intention of injury was categorized as intentional and unintentional. Sports-related concussions were identified and categorized as a binary variable (yes vs. no) using the Association of Public Health Epidemiologists in Ontario (APHEO) definitions of different sports injuries.^42^ Using the definitions stated above, patients in the ED were additionally stratified into those with an MVC-related concussion and those with a sports-related concussion Supplementary Table S5.

The outcome variable was the presence of a comorbid neck injury at the time of the ED visit. This was identified using ICD-10-CA codes S12 to S17 and S19. Superficial injuries of the neck (S10), open wounds of the neck (S11), and traumatic amputation at neck level (S18) were excluded to align with our hypothesis, namely that there may be higher rates of comorbid neck injury among females due to differing neck strength between males and females. Since the ICD-10-CA codes S10 an S11 do not reflect this notion, they were not included in this case definition of comorbid neck injury.

### Statistical analyses

Frequencies and rates of comorbid neck injuries per 100,000 concussion-related ED visits during the study period were determined, stratified by sex and cause of injury (MVC and sports, as defined above regarding injury-related variables). A multivariable logistic regression model was used to measure the odds ratios (ORs) and 95% confidence interval (CI, Wald) for each of the covariates. All covariates except for age were included as linear terms in the model. Age was modeled as a cubic polynomial, that is, terms for age, age^2^, and age^3^ were included in the model. This is analogous to using a polynomial regression.^43,44^ Since the curves for the association between age and rate of comorbid neck injury were different for male and female patients, the model included an interaction term between the effects of age and sex. Due to the fact that the sex and age interaction in the cubic polynomial can be difficult to interpret, a more straightforward depiction of the effect for age and sex was reported by calculating the ORs for each given age group and sex. Tests of significance were two-sided tests with significance levels of 95%. All analyses were conducted using SAS 9.3,^45^ and figures in this article were created using R 3.4.1.^46^

## Results

Between fiscal years 2002/2003 and 2011/2012, 90,661 unique individuals were coded with a concussion-related ED visit in Ontario. Patients with missing data of interest (*i.e.*, mechanism of injury, intention of injury, rurality, or income quintile; *N* = 486) were excluded, leaving 90,175 patients for inclusion, 58% of whom were males. By cause of injury, 8,134 patients (53% males) with TBI were in the ED due to an MVC and 30,474 (70% males) were in the ED due to a sports injury Supplementary Fig. 1.

Table 1 and Supplementary Table S1 present the sociodemographic and injury-related characteristics of the three samples examined. The rate of comorbid neck injury per 100,000 concussion-related ED visits was higher among females (4,333 vs. 2,995/100,000). This pattern was also observed when the data were stratified by cause of injury (11,978 vs. 8,759/100,000 in MVC-related concussions and 4,207 vs. 2,794/100,000 in sports-related concussions). The rate of comorbid neck injury was 2.89 times higher for patients with an MVC-related concussion compared with all concussion-related ED visits (2.92 times higher for males and 2.76 times higher for females). From fiscal years 2002/2003 to 2011/2012, concussion-related diagnoses in the ED setting increased by 19% among males and 83% among females while the rate of comorbid neck injury remained relatively stable. However, over this time period, the rate of comorbid neck injury among patients injured *via* MVC increased by 39% among males and 29% among females. Finally, for intentional injuries, the rate of comorbid neck injury was 2.70 times higher for females than for males, whereas only 1.39 times higher for unintentional injuries.

**Table 1.** Sociodemographic and Injury Characteristics of Patients with a First Concussion-Related Emergency Department Visit and Rate of Comorbid Neck Injuries per 100,000 Concussion-Related Emergency Department Visits in Ontario, Canada, 2002/2003–2011/2012, by Sex

	*All concussions*						*MVC-related concussions*						*Sports-related concussions*					
	*Rate of comorbid neck injuries*						*Rate of comorbid neck injuries*						*Rate of comorbid neck injuries*					
	*Overall*		*Males*^a^		*Females*^a^		*Overall*		*Males*^a^		*Females*^a^		*Overall*		*Males*^a^		*Females*^a^	
*Characteristic*	*Total No. of concussions (%)*	*Rate per 100,000*	*Total No. of concussions (%)*	*Rate per 100,000*	*Total No. of concussions (%)*	*Rate per 100,000*	*Total No. of concussions (%)*	*Rate per 100,000*	*Total No. of concussions (%)*	*Rate per 100,000*	*Total No. of concussions (%)*	*Rate per 100,000*	*Total No. of concussions (%)*	*Rate per 100,000*	*Total No. of concussions (%)*	*Rate per 100,000*	*Total No. of concussions (%)*	*Rate per 100,000*
Overall	90,175	3,555	52,418	2,995	37,757	4,333	8,134	10,266	4,327	8,759	3,807	11,978	30,474	3,219	21,298	2,794	9,176	4,207
Fiscal year of ED discharge																		
2002/2003	8,571 (9.5)	3,407	5,488 (10.5)	3,098	3,083 (8.2)	3,957	808 (9.9)	7,921	462 (10.7)	6,926	346 (9.1)	9,249	2,885 (9.5)	2,946	2,178 (10.2)	2,938	707 (7.7)	2,970
2003/2004	7,616 (8.4)	3,768	4,853 (9.3)	3,379	2,763 (7.3)	4,452	755 (9.3)	8,742	438 (10.1)	7,763	317 (8.3)	10,095	2,674 (8.8)	3,889	2,021 (9.5)	3,365	653 (7.1)	5,513
2004/2005	8,389 (9.3)	4,077	5,177 (9.9)	3,342	3,212 (8.5)	5,262	787 (9.7)	10,292	437 (10.1)	8,467	350 (9.2)	12,571	2,831 (9.3)	3,709	2,096 (9.8)	2,815	735 (8.0)	6,259
2005/2006	7,902 (8.8)	4,164	4,842 (9.2)	3,697	3,060 (8.1)	4,902	737 (9.1)	10,991	415 (9.6)	10,602	322 (8.5)	11,491	2,684 (8.8)	3,614	1,927 (9.0)	3,166	757 (8.2)	4,756
2006/2007	8,034 (8.9)	3,199	4,786 (9.1)	2,591	3,248 (8.6)	4,095	820 (10.1)	11,829	478 (11.0)	9,623	342 (9.0)	14,912	2,586 (8.5)	2,359	1,832 (8.6)	2,183	754 (8.2)	2,785
2007/2008	8,472 (9.4)	3,458	4,968 (9.5)	2,899	3,504 (9.3)	4,252	789 (9.7)	9,632	419 (9.7)	7,876	370 (9.7)	11,622	2,719 (8.9)	3,604	1,910 (9.0)	3,037	809 (8.8)	4,944
2008/2009	8,702 (9.7)	3,493	4,873 (9.3)	2,811	3,829 (10.1)	4,361	767 (9.4)	10,952	387 (8.9)	8,527	380 (10.0)	13,421	2,797 (9.2)	3,432	1,929 (9.1)	2,799	868 (9.5)	4,839
2009/2010	9,863 (10.9)	3,326	5,343 (10.2)	2,527	4,520 (12.0)	4,270	829 (10.2)	10,615	400 (9.2)	8,250	429 (11.3)	12,821	3,315 (10.9)	3,047	2,187 (10.3)	2,378	1,128 (12.3)	4,344
2010/2011	10,452 (11.6)	3,368	5,564 (10.6)	2,858	4,888 (12.9)	3,948	883 (10.9)	10,646	434 (10.0)	9,908	449 (11.8)	11,359	3,563 (11.7)	2,779	2,317 (10.9)	2,719	1,246 (13.6)	2,889
2011/2012	12,174 (13.5)	3,466	6,524 (12.4)	2,836	5,650 (15.0)	4,195	959 (11.8)	10,845	457 (10.6)	9,628	502 (13.2)	11,952	4,420 (14.5)	3,054	2,901 (13.6)	2,620	1,519 (16.6)	3,884
Income quintile																		
1	15,765 (17.5)	3,692	8,919 (17.0)	3,341	6,846 (18.1)	4,148	1,649 (20.3)	10,673	883 (20.4)	9,740	766 (20.1)	11,749	3,982 (13.1)	3,039	2,772 (13.0)	2,670	1,210 (13.2)	3,884
2	16,684 (18.5)	3,698	9,596 (18.3)	3,178	7,088 (18.8)	4,402	1,586 (19.5)	9,899	840 (19.4)	9,643	746 (19.6)	10,188	5,092 (16.7)	3,201	3,555 (16.7)	2,785	1,537 (16.8)	4,164
3	17,848 (19.8)	3,782	10,337 (19.7)	2,970	7,511 (19.9)	4,899	1,649 (20.3)	11,704	899 (20.8)	8,343	750 (19.7)	15,733	5,971 (19.6)	3,668	4,174 (19.6)	3,019	1,797 (19.6)	5,175
4	19,683 (21.8)	3,404	11,656 (22.2)	2,805	8,027 (21.3)	4,273	1,685 (20.7)	9,555	895 (20.7)	7,374	790 (20.8)	12,025	7,264 (23.8)	3,125	5,143 (24.1)	2,839	2,121 (23.1)	3,819
5	20,195 (22.4)	3,278	11,910 (22.7)	2,796	8,285 (21.9)	3,971	1,565 (19.2)	9,457	810 (18.7)	8,765	755 (19.8)	10,199	8,165 (26.8)	3,074	5,654 (26.5)	2,653	2,511 (27.4)	4,022
Rurality																		
Yes	16,621 (18.4)	4,001	9,592 (18.3)	3,482	7,029 (18.6)	4,709	1,367 (16.8)	9,510	770 (17.8)	8,571	597 (15.7)	10,720	5,754 (18.9)	3,737	3,948 (18.5)	3,217	1,806 (19.7)	4,873
No	73,554 (81.6)	3,455	42,826 (81.7)	2,886	30,728 (81.4)	4,247	6,767 (83.2)	10,418	3,557 (82.2)	8,800	3,210 (84.3)	12,212	24,720 (81.1)	3,099	17,350 (81.5)	2,697	7,370 (80.3)	4,043
Mechanism of injury																		
Fall	35,949 (39.9)	2,737	18,182 (34.7)	2,101	17,767 (47.1)	3,388							6,912 (22.7)	3,328	4,216 (19.8)	2,419	2,696 (29.4)	4,748
Struck by/against	35,593 (39.5)	2,944	23,429 (44.7)	2,770	12,164 (32.2)	3,280							18,368 (60.3)	3,430	13,281 (62.4)	3,087	5,087 (55.4)	4,325
MVC	8,134 (9.0)	10,266	4,327 (8.3)	8,759	3,807 (10.1)	11,978							512 (1.7)	3,516	393 (1.8)	NR^b^	119 (1.3)	*n* < 6
Other transportation	7,313 (8.1)	3,596	4,529 (8.6)	2,716	2,784 (7.4)	5,029							4,651 (15.3)	NR^b^	3,386 (15.9)	1,949	1,265 (13.8)	NR^b^
Other	3,186 (3.5)	2,385	1,951 (3.7)	1,896	1,235 (3.3)	3,158							31 (0.1)	*n* < 6	22 (0.1)	*n* < 6	9 (0.1)	0
Intention of injury																		
Unintentional	85,138 (94.4)	3,641	48,530 (92.6)	3,114	36,608 (97.0)	4,341												
Intentional	5,037 (5.6)	2,104	3,888 (7.4)	1,517	1,149 (3.0)	4,091												
Sports-related injury																		
Yes	30,474 (33.8)	3,219	21,298 (40.6)	2,794	9,176 (24.3)	4,207												
No	59,701 (66.2)	3,727	31,120 (59.4)	3,133	28,581 (75.7)	4,374												

Table on age is included in Supplementary Tables S1–S4.

*n* < 6—cell size less than 6.

^a^ Unadjusted OR for sex (female vs. male) was 1.47 (95% CI 1.37–1.57) for all concussions, 1.42 (95% CI 1.23–1.64) for MVC concussions, and 1.53 (95% CI 1.34–1.74) for concussions due to sports injuries. Note: adjusted OR and 95% CI for sex can be found in Figure 2.

^b^ Data are NR due to residual disclosure of small cell size (*n* < 6).

CI, confidence interval; ED, emergency department; MVC, motor vehicle collision; NR, not reported; OR, odds ratio.

**FIG. 1.**
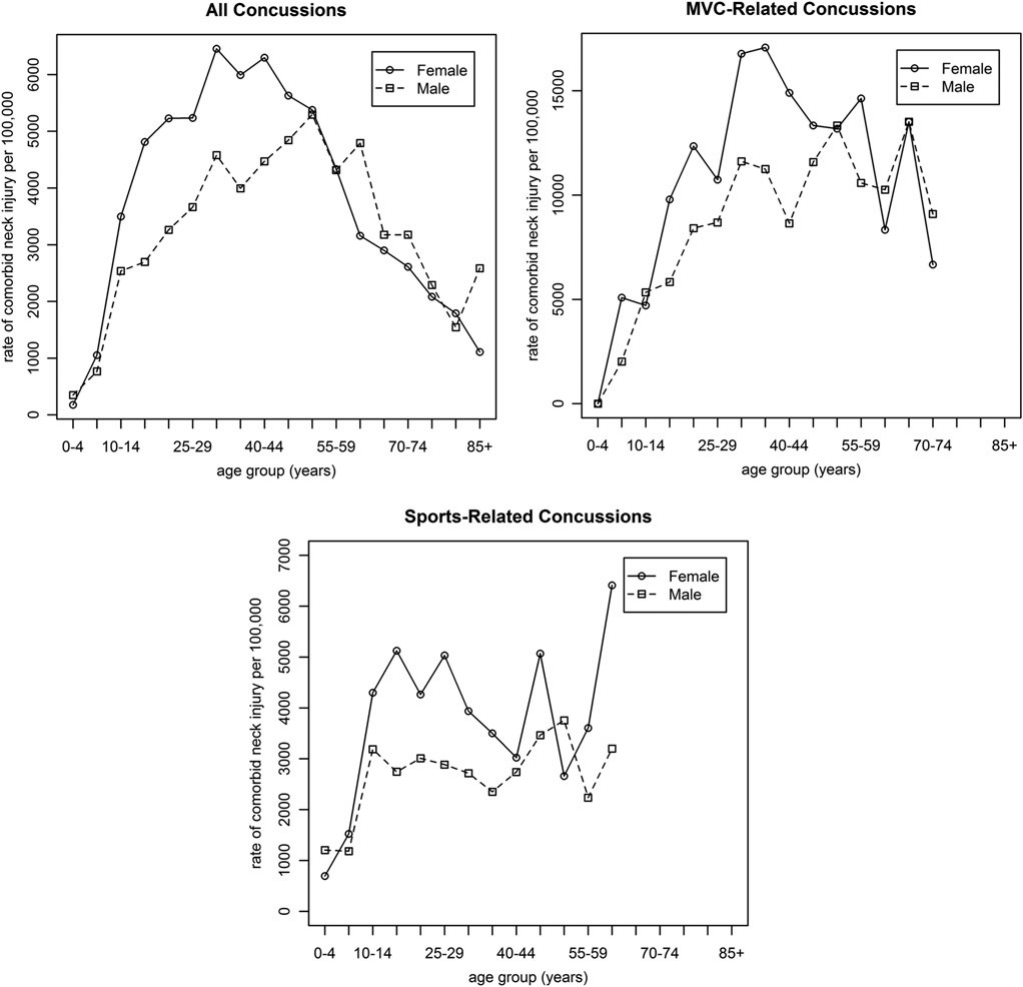
Rates of comorbid neck injury per 100,000 concussion-related emergency department visits in Ontario, Canada, 2002/2003–2011/2012, by 5-year age groups, sex, and cause of injury. The rates of neck injuries for the age group 70–74 years for MVC-related concussions and age group 60–64 years for sports-related concussions were not reported due to small sample size. MVC, motor vehicle collision.

Figure 1 presents the rate of comorbid neck injuries per 100,000 patients with a concussion-related ED visit by 5-year age groups, sex, and cause of injury (MVC and sports). Females had a higher rate of neck injury compared with males between the ages of 10 and 50 years, after which both males and females had similar rates of comorbid neck injury.

### Polynomial logistic regression model

**FIG. 2.**
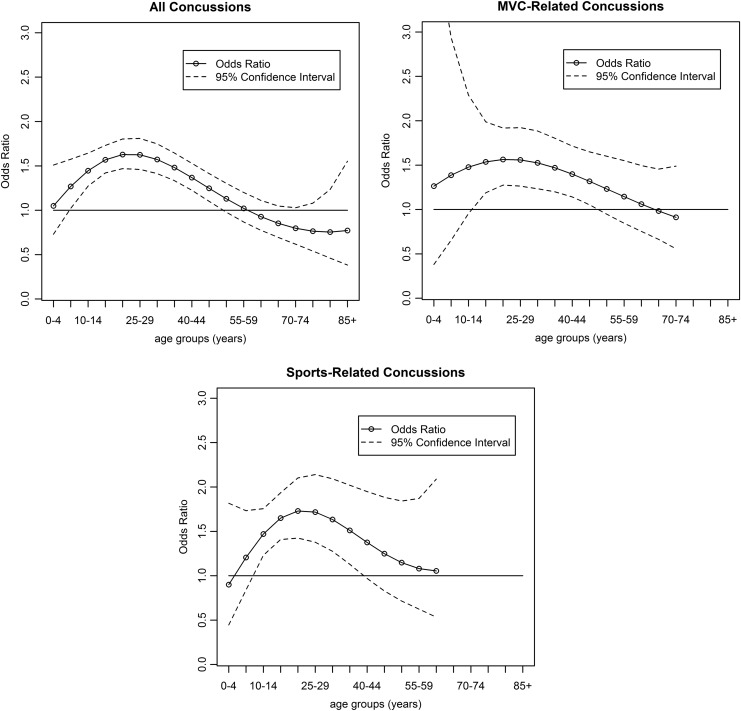
Odds ratio and 95% confidence interval of sex (reference = male) of comorbid neck injury among patients with a concussion-related emergency department visits in Ontario, Canada, 2002/2003–2011/2012, by age groups and cause of injury. The rates of neck injuries for the age group 70–74 years for MVC-related concussions and age group 60–64 years for sports-related concussions were not reported due to small sample size.

Figure 2 and Supplementary Table S2 display the results of the cubic polynomial logistic regression model, which produced a moving OR for sex (with males as the referent population) by each 5-year age group. Females had significantly higher odds of sustaining a comorbid neck injury between the ages of 5–49 years for all concussion-related ED visits, 15–49 years for MVC-related concussion ED visits, and 10–39 years for sports-related concussion ED visits, adjusting for all other variables. Patients between the ages of 20 and 24 years had the highest odds of sustaining a comorbid neck injury (OR = 1.56 [95% CI: 1.47–1.80]), including those injured in an MVC (OR = 1.26 [95% CI: 1.27–1.92]) and in sport activities (OR = 1.73 [95% CI: 1.42–2.10]). The overall significance of sex in this model was found to be highly significant (*p* < 0.0001) for all three populations using the log-likelihood test.

The ORs and 95% Wald CI of comorbid neck injuries for all other covariates included in this model are presented in Table 2. Among all patients with a concussion-related ED visit, the odds of a comorbid neck injury among those injured in an MVC was 3.40 times higher than the odds of the same patient who experienced a fall (95% CI: 3.08–3.76). Other covariates found to be significant for this population included rurality (OR = 1.21 [95% CI: 1.11–1.33]), sports injury (OR = 1.24 [95% CI: 1.13–1.36]), fiscal year of ED discharge (OR = 0.98 [95% CI: 0.97–0.99]), and intent of injury (OR = 0.71 [95% CI: 0.57–0.87]). Among patients with a sports-related injury, rurality (OR = 1.22 [95% CI: 1.04–1.43]) and fiscal year of ED discharge (OR = 0.97 [95% CI: 0.95–0.99]) were significant, with similar trends to that identified in the entire population. No other covariates included in the model for MVC-related concussions were statistically significant.

**Table 2.** Logistic Regression Model Examining the Odds of Comorbid Neck Injury for Other Covariates Among Patients with a First Concussion-Related Emergency Department Visit in Ontario, Canada, 2002/2003–2011/2012

		*All concussions*			*MVC-related concussions*			*Sports-related concussions*		
*Effect*	*Category*	*OR*	*95% CI*	p	*OR*	*95% CI*	p	*OR*	*95% CI*	p
Sex^a^		Due to interaction between sex and age, there is not a single OR for the variable sex								
Age^b^		Due to interaction between sex and age, there is not a single OR for the variable age								
Mechanism of injury	Fall	Ref	Ref	Ref				Ref	Ref	Ref
	MVC	3.401	3.08–3.76	0.000				1.07	0.65–1.75	0.788
	Struck by/against	1.059	0.96–1.17	0.247				1.037	0.89–1.21	0.650
	Other transportation	1.134	0.98–1.31	0.088				0.642	0.51–0.82	0.000
	Other	0.836	0.66–1.06	0.142				3.092	0.93–10.32	0.066
Income^c^		0.987	0.96–1.01	0.305	0.976	0.93–1.03	0.350	0.995	0.95–1.04	0.835
Rurality	No	Ref	Ref	Ref	Ref	Ref	Ref	Ref	Ref	Ref
	Yes	1.214	1.11–1.33	0.000	0.965	0.79–1.18	0.725	1.221	1.04–1.43	0.012
Sports injury	No	Ref	Ref	Ref						
	Yes	1.235	1.13–1.36	0.000						
Fiscal year of ED discharge^c^		0.982	0.97–0.99	0.003	1.013	0.99–1.04	0.299	0.973	0.95–0.99	0.012
Intention of injury	Unintentional	Ref	Ref	Ref						
	Intentional	0.705	0.57–0.87	0.001						

^a^ OR and 95% CI of sex, by 5-year age group, can be found in Figure 2 and Supplementary Table S2.

^b^ OR and 95% CI of age, by 5-year age group and sex, can be found in Supplementary Tables S3 and S4.

^c^ Linear trend as an ordinal variable.

Ref, referent group.

## Discussion

This study provided evidence that females with a concussion-related ED visit between the ages of 5 and 49 years had a significantly higher rate of comorbid neck injury than males across all injury contexts (*i.e.*, all concussions, MVC, and sports). This was identified using interaction terms between sex and age, where it was assumed that age did not follow a linear trend, but rather, a cubic trend. This was a unique approach to modeling that, to the best of our knowledge, has not been widely used in other concussion-related research.^26,27^ Future studies on concussions should consider using similar linear and nonlinear sex and age interaction terms across the life span in their models and analysis.

Although results from this study contradict earlier studies by Fujii et al.^26^ and Hasler et al.,^27^ these inconsistencies may be attributed to differences in the methodology and sample size, or the selection of patients with differing mechanisms of injury (*e.g.*, trauma center patients). Specifically, the above studies analyzed the rate of cervical spine injury in major trauma patients and reported that females had a lower rate of neck injury (OR = 0.90 [95% CI: 0.84–0.96] and OR = 0.91 [95% CI: 0.86–0.96], respectively) compared with males.^26,27^ This study focused on the patients in the ED setting with concussions that are of mild injury severity. As such, findings from our study support increased attention in the ED setting for a potential neck injury among females, aged 5–49 years, with a concussion (particularly those between 15 and 49 years of age with an MVC-related concussion and those between 10 and 39 years of age for a sports-related concussion), to allow for early intervention and treatment. In particular, further work is required to determine the potential anatomical and biological differences that underlie the apparent increased rate of comorbid neck injuries among females with a concussion. Given that neck injury in TBI has been linked to persistent postconcussive symptoms such as dizziness, unsteadiness, gaze stability, and head–eye coordination,^12–16,47–49^ future efforts should explore sex-specific differences in these postconcussive symptoms and its impact on injury severity, recovery time, intervention, and the rehabilitation process to support appropriate care and management for patients.^50,51^

This study found that females, aged 15–49 years, who were injured in an MVC had significantly higher odds of comorbid neck injury, which may reflect the fact that individuals are not legally allowed to drive until the age of 16 in Ontario.^52^ Similarly, our results indicated that females, aged 10–39 years, who were injured in a sports-related activity, had significantly higher odds of a comorbid neck injury compared with males, possibly reflecting a participation in sports among younger adults.^53^ These findings encourage additional research to understand how best to prevent head and neck injuries for this population, including sex-specific prevention or harm reduction strategies such as improving protective equipment in sports for both males and females, and safety measures for motor vehicle drivers, occupants, or cyclists.

Data on other hypothesized variables, including mechanism and cause of injury (*i.e.*, MVC, sports injuries) and residence in rural neighborhoods, were significantly associated with neck injury comorbidity in all concussion-related ED visits, holding all other variables constant. This is consistent with previous studies, which reported higher odds of sustaining a neck injury in MVC and sports-related incidents compared with all concussion-related injuries.^17,21,22,54^ Furthermore, the results of our study suggest that females have a higher risk of sustaining a comorbid neck injury across all causes of injury. Additionally, our study indicates that although overall rates for neck injury were lower for intentional injuries, females who suffer a concussion from an intentional injury may have a rate of comorbid neck injury that is higher than that of males. These findings further support a sex-specific approach to study and analyze concussion data, particularly females whose injury is a result of assault/violence.

### Strengths and limitations

One of the main strengths of this study was the use of a population-based data set, the NACRS, from a publicly funded health system to identify all ED visits in the province of Ontario, home to ∼40% of the Canadian population.^55^ Use of these data eliminated some of the inherent issues previously identified (*i.e.*, insurance bias, attrition) in studying neck injury comorbidity in concussion-related ED visits within nonpublicly funded health care systems. Our study is the first, to the best of our knowledge, to highlight the critical influence of age and sex on neck injury comorbidity in concussion-related ED visits across the life span. We used a novel method of modeling the variable age with a cubic trend and as an interaction term with sex, whereas previous related studies assumed linear trends for these covariates.^26,27^ Such an approach is the optimal methodology in situations where male and female sex exerts differential influence on the outcome variable (in this instance, neck injury comorbidity) across different age groups. Finally, previous studies included patients with TBI that range in severity from mild to severe^26,27^; this study reduced the risk of selection bias by only analyzing patients with concussion-related ED visits and mild severity.

A recognized limitation of this study was that the number of patients with a first concussion may be underestimated, as the data set excluded patients with a concussion who (a) did not visit the ED, but instead sought care from a family physician or an acute care setting; (b) did not seek any care at all; or (c) were not diagnosed with an ICD-10-CA S06.0 code. Therefore, this study may suffer from potential selection bias, as only data on ED visits were included. We also adopted a conservative ICD-10-CA case definition for concussion injury, which was also used by other investigators.^56^ As a result, it is possible that concussions may have been missed in the emergency room or were coded under other ICD-10-CA codes such as “unspecified injuries to the head.” An additional limitation was that our study only included patients with their first concussion-related ED visit between 2002/2003 and 2011/2012; therefore, we were unable to determine if they had concussion-related visits before the study period. Limitations associated with health administrative data were also recognized (*e.g.*, variables of interest may have been unavailable, missed diagnosis due to coding). Also, it is acknowledged that this study examined age using 5-year age groups, which may not be adequate to detect differences in younger populations. Furthermore, it was not possible to determine from health administrative data how these findings may have been influenced by gender differences in reporting concussion.^57–59^ Finally, we recognize that the literature on concussion in older adults is understudied and our finding may reflect that lack of concussion protocols and complexity regarding the identification of comorbidities in this population.^60^ Despite these limitations, elucidation of sex differences in neck injury comorbidity across the life span could have a tremendous impact on how neck injuries are viewed and managed among patients with a concussion in the future.

## Conclusions

This study showed an increased risk of comorbid neck injury in females with a concussion-related ED visit, which has strong implications for patients, clinicians, and decision-makers. While there has been increased awareness of concussions in the media in recent years, especially sports-related concussions,^54^ this study supports the hypothesis that neck injuries and concussions, which have similar symptoms,^12–16^ frequently co-occur.^17^ Therefore, our findings support the need for increased consideration of comorbid neck injuries to allow for early intervention, particularly for females, who have been found to be most at risk for persistent symptoms.^10,11^ Most importantly, the results of this study suggest that the biological differences between males and females,^19,20^ which determine the risk of comorbid neck injury, are age-dependent. This study showed a clear interaction between age and sex and, therefore, it is crucial to consider linear and nonlinear sex and age interactions across the life span of patients in future studies on concussions.

## Supplementary Material

Supplemental data

Supplemental data

Supplemental data

Supplemental data

Supplemental data

Supplemental data

## Disclaimer

This study was supported through the provision of data by the Institute for Clinical Evaluative Sciences (ICES) and Cancer Care Ontario (CCO) and through funding support to ICES from an annual grant by the Ministry of Health and Long-Term Care (MOHLTC) and the Ontario Institute for Cancer Research (OICR). The opinions, results, and conclusions reported in this article are those of the authors and are independent of the funding sources. No endorsement by ICES, CCO, OICR, or the Government of Ontario is intended or should be inferred. Parts of this material are based on data and information compiled and provided by CIHI. However, the analyses, conclusions, opinions, and statements expressed herein are those of the author and not necessarily those of CIHI.
