# Supplementary material for: Neck Injury Comorbidity in Concussion-Related Emergency Department Visits: A Population-Based Study of Sex Differences Across the Life Span
Source: J Womens Health (Larchmt). 2019 Apr 22;28(4):473–82. doi: 10.1089/jwh.2018.7282 (PMC6482894; doi:10.1089/jwh.2018.7282)
Supplement: Supplemental data [file Supp_Table5.pdf]

Supplementary Table S5. International Statistical Classification of Diseases and Related Health Problems, Tenth Revision, Canada (ICD-10-CA) Used to Define Concussion, Neck Injury, Mechanism of Injury, Intention of Injury, and Sports Injury

| | |
|---|---|
| Concussion | S06.0 |
| Injuries to the neck | (S12–S17), S19 |
| Case definitions used for mechanism of injury | |
| Fall | (W00–W19), X80, Y01, Y30 |
| Struck by/against | (W20–W22), (W50–W52), X79, Y00, Y04, Y35.3, Y29 |
| MVC | (V30–V79 [only 0.4–0.9]), (V81–V82 [only 0.1]), (V83–V86 [only 0.0–0.3]), (V20–V28 [only 0.3–0.9]), V29 (only 0.4–0.9), (V12–V14 [only 0.3–0.9]), V19 (only 0.4–0.6), (V02–V04 [only 0.1–0.9]), V09.2, V80 (only 0.3–0.5), V87 (only 0.0–0.8), V89.2 |
| Other transportation | |
| Other pedal cycling | (V10, V11, V15, V16, V17, V18), (V12–V14 [only 0.0–0.2]), V19 (only 0.0–0.3, 0.8–0.9) |
| Other pedestrian | (V01, V05, V06), (V02–V04 [only 0.0–0.3]), V09 (only 0.0, 0.1, 0.3, 0.9) |
| Other land transport | (V20–V28 [only 0.0–0.2]), V29 (only 0.0–0.3), (V30–V79 [only 0.0–0.3]), V80 (only 0.0–0.2 and 0.6–0.9), (V81–V82 [not 0.1]), (V83–V86 [only 0.4–0.9]), V87.9, V88 (0.0–0.9), V89 (only in 0.0,0.1,0.3,0.9), X82, Y03, Y32 |
| Other transport | (V90–V99), Y361, U011 |
| Other | |
| E.g., drowning, fire/flame, and cut/pierce | All other injury-related ICD-10-CA codes |
| Case definitions used for intention of injury | |
| Unintentional | |
| Unintentional | (V01–X59), Y85, Y86 |
| Undetermined | (Y10–Y34), Y87.2, Y89.9 |
| Intentional | |
| Assault | (X85–Y09), Y87.1, (U01–U02) |
| Suicide | (X60–X84), Y87.0, U03 |
| Legal/war | Y35, Y36, Y89.0-Y89.1 |
| Case definitions used for sports injuries | |
| Baseball | W22.05, W51.05 |
| Hit by ball | W21.00 |
| Hit by bat | W21.01 |
| Cycling | V10–V19 |
| Fall involving rollerblade/scooter/skateboard | W02.02, W02.03, W02.08 |
| Football/rugby | W22.03, W51.03 |
| Hockey | W21.02, W21.03, W22.02, W51.02 |
| Ice skates | W02.00 |
| Playground equipment | W09 |
| Pool and natural water swimming/diving/drowning | W16, (W67–W74) |
| Ski/snowboard | W02.01,W02.04, W22.00, W51.00 |
| Soccer | W22.04, W51.04 |
| Tobogganing | W22.01, W51.01 |
| Recreational boating | (V90–V94 [only 0.2–0.8]) |
| ATV/snowmobile | V86 |
| Other sports-related injuries | W02.08, W21.08, W21.09, W22.07, W51.07 |

Notes on injury severity: Severity grouped into mild, moderate, and severe by ICD-10-CA code, as per Abbreviated Injury Scale (AIS). Injuries classified as Unknown occurred when ICD-10-CA code was not specific enough.
