# Supplementary material for: Neck Injury Comorbidity in Concussion-Related Emergency Department Visits: A Population-Based Study of Sex Differences Across the Life Span
Source: J Womens Health (Larchmt). 2019 Apr 22;28(4):473–82. doi: 10.1089/jwh.2018.7282 (PMC6482894; doi:10.1089/jwh.2018.7282)
Supplement: Supplemental data [file Supp_Fig1.pdf]

Supplementary Data

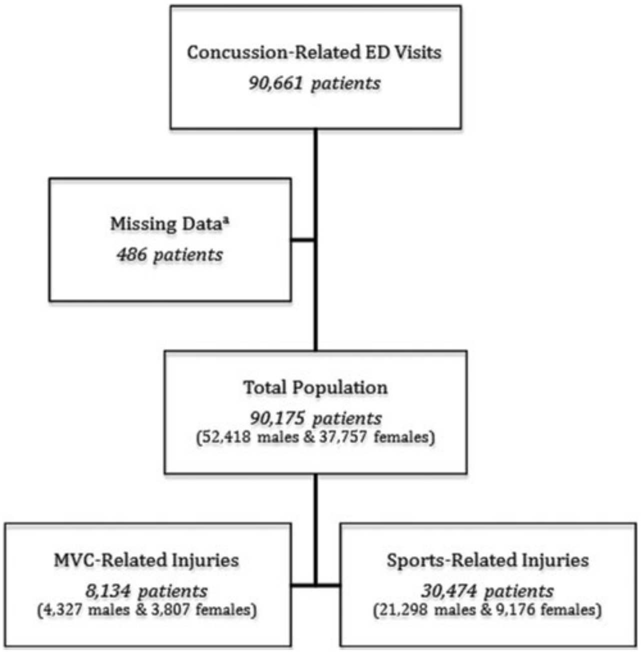

SUPPLEMENTARY FIG. S1. Flow diagram of all eligible patients with a first concussion-related emergency department visit in Ontario, Canada, 2002/2003–2011/2012. [a]Missing data: 470 patients were removed due to missing data for income quantile. One hundred twenty-one patients were removed due to missing data for rurality. Sixteen patients were removed due to missing data for mechanism of injury. Sixteen patients were removed due to missing data for intention of injury. Total number of patients removed due to missing data: 486.
