# Supplementary material for: Neck Injury Comorbidity in Concussion-Related Emergency Department Visits: A Population-Based Study of Sex Differences Across the Life Span
Source: J Womens Health (Larchmt). 2019 Apr 22;28(4):473–82. doi: 10.1089/jwh.2018.7282 (PMC6482894; doi:10.1089/jwh.2018.7282)
Supplement: Supplemental data [file Supp_Table1.pdf]

Supplementary Table S1. Number of First Concussion-Related Emergency Department Visits and Rate of Comorbid Neck Injuries Per 100,000 Concussion-Related Emergency Department Visits in Ontario, Canada, 2002/2003–2011/2012, by Age Groups and Sex

	All concussions						*MVC-related concussions*						*Sports-related concussions*					
	Rate of comorbid neck injuries						*Rate of comorbid neck injuries*						*Rate of comorbid neck injuries*					
	Overall		*Males*		*Females*		*Overall*		*Males*		*Females*		*Overall*		*Males*		*Females*	
Characteristic	*Total No. of concussions (%)*	*Rate per 100,000*	*Total No. of concussions (%)*	*Rate per 100,000*	*Total No. of concussions (%)*	*Rate per 100,000*	*Total No. of concussions (%)*	*Rate per 100,000*	*Total No. of concussions (%)*	*Rate per 100,000*	*Total No. of concussions (%)*	*Rate per 100,000*	*Total No. of concussions (%)*	*Rate per 100,000*	*Total No. of concussions (%)*	*Rate per 100,000*	*Total No. of concussions (%)*	*Rate per 100,000*
Overall	90,175	3,555	52,418	2,995	37,757	4,333	8,134	10,266	4,327	8,759	3,807	11,978	30,474	3,219	21,298	2,794	9,176	4,207
Age group, years																		
0–4	3,701 (4.1)	270	2,002 (3.8)	NR[a]	1,699 (4.5)	$n<6$	54 (0.7)	0	29 (0.7)	0	25 (0.7)	0	310 (1.0)	$n<6$	166 (0.8)	$n<6$	144 (1.6)	$n<6$
5–9	6,234 (6.9)	866	4,047 (7.7)	766	2,187 (5.8)	1,052	158 (1.9)	$n<6$	99 (2.3)	$n<6$	59 (1.5)	$n<6$	2,261 (7.4)	1,283	1,605 (7.5)	1,184	656 (7.1)	1,524
10–14	16,560 (18.4)	2,826	11,583 (22.1)	2,538	4,977 (13.2)	3,496	395 (4.9)	5,063	225 (5.2)	5,333	170 (4.5)	4,706	9,761 (32.0)	3,463	7,342 (34.5)	3,187	2,419 (26.4)	4,299
15–19	18,639 (20.7)	3,482	11,720 (22.4)	2,696	6,919 (18.3)	4,813	1,569 (19.3)	7,712	823 (19.0)	5,832	746 (19.6)	9,786	9,816 (32.2)	3,454	6,889 (32.3)	2,744	2,927 (31.9)	5,125
20–24	8,147 (9.0)	4,087	4,722 (9.0)	3,261	3,425 (9.1)	5,226	1,193 (14.7)	10,226	642 (14.8)	8,411	551 (14.5)	12,341	2,189 (7.2)	3,426	1,462 (6.9)	3,010	727 (7.9)	4,264
25–29	5,509 (6.1)	4,338	3,140 (6.0)	3,662	2,369 (6.3)	5,234	826 (10.2)	9,564	472 (10.9)	8,686	354 (9.3)	10,734	1,393 (4.6)	3,589	936 (4.4)	2,885	457 (5.0)	5,033
30–34	4,619 (5.1)	5,434	2,512 (4.8)	4,578	2,107 (5.6)	6,455	651 (8.0)	13,978	353 (8.2)	11,615	298 (7.8)	16,779	1,007 (3.3)	3,178	626 (2.9)	2,716	381 (4.2)	3,937
35–39	4,499 (5.0)	4,957	2,329 (4.4)	3,993	2,170 (5.7)	5,991	616 (7.6)	13,961	329 (7.6)	11,246	287 (7.5)	17,073	896 (2.9)	2,790	553 (2.6)	2,351	343 (3.7)	3,499
40–44	4,585 (5.1)	5,387	2,282 (4.4)	4,470	2,303 (6.1)	6,296	626 (7.7)	11,661	324 (7.5)	8,642	302 (7.9)	14,901	875 (2.9)	2,857	511 (2.4)	2,740	364 (4.0)	3,022
45–49	4,133 (4.6)	5,250	1,983 (3.8)	4,841	2,150 (5.7)	5,628	570 (7.0)	12,456	285 (6.6)	11,579	285 (7.5)	13,333	729 (2.4)	4,115	433 (2.0)	3,464	296 (3.2)	5,068
50–54	3,503 (3.9)	5,338	1,587 (3.0)	5,293	1,916 (5.1)	5,376	445 (5.5)	13,258	225 (5.2)	13,333	220 (5.8)	13,182	481 (1.6)	3,326	293 (1.4)	3,754	188 (2.0)	2,660
55–59	2,821 (3.1)	4,325	1,298 (2.5)	4,314	1,523 (4.0)	4,334	353 (4.3)	12,465	189 (4.4)	10,582	164 (4.3)	14,634	290 (1.0)	2,759	179 (0.8)	$n<6$	111 (1.2)	$n<6$
60–64	2,036 (2.3)	3,929	960 (1.8)	4,792	1,076 (2.8)	3,160	225 (2.8)	9,333	117 (2.7)	10,256	108 (2.8)	8,333	203 (0.7)	4,433	125 (0.6)	$n<6$	78 (0.9)	$n<6$
65–69	1,486 (1.6)	3,028	693 (1.3)	3,175	793 (2.1)	2,900	148 (1.8)	13,514	74 (1.7)	13,514	74 (1.9)	13,514	135 (0.4)	$n<6$	88 (0.4)	$n<6$	47 (0.5)	0
70–74	1,180 (1.3)	2,881	567 (1.1)	3,175	613 (1.6)	2,610	115 (1.4)	7,826	55 (1.3)	$n<6$	60 (1.6)	$n<6$	64 (0.2)	0	40(0.2)	0	24 (0.3)	0
75–79	1,013 (1.1)	2,172	437 (0.8)	2,288	576 (1.5)	2,083	100 (1.2)	7,000	45 (1.0)	$n<6$	55 (1.4)	$n<6$	40 (0.1)	0	28 (0.1)	0	12 (0.1)	0
80–84	827 (0.9)	1,693	324 (0.6)	$n<6$	503 (1.3)	NR[a]	62 (0.8)	$n<6$	29 (0.7)	$n<6$	33 (0.9)	$n<6$	17 (0.1)	0	NR[a]	0	$n<6$	0
85+	683 (0.8)	1,611	232 (0.4)	NR[a]	451 (1.2)	$n<6$	28 (0.3)	$n<6$	12 (0.3)	$n<6$	16 (0.4)	0	7 (0.0)	$n<6$	NR[a]	$n<6$	NR[a]	0

$n<6$—cell size less than 6.

[a]Data are NR due to residual disclosure of small cell size ($n<6$).

ED, emergency department; MVC, motor vehicle collision; NR, not reported.
