# Supplementary material for: Neck Injury Comorbidity in Concussion-Related Emergency Department Visits: A Population-Based Study of Sex Differences Across the Life Span
Source: J Womens Health (Larchmt). 2019 Apr 22;28(4):473–82. doi: 10.1089/jwh.2018.7282 (PMC6482894; doi:10.1089/jwh.2018.7282)
Supplement: Supplemental data [file Supp_Table4.pdf]

Supplementary Table S4. Logistic Regression Model Examining the Odds of Comorbid Neck Injury for Age Among Male Patients With a First Concussion-Related Emergency Department Visit in Ontario, Canada, 2002/2003–2011/2012, by 5-Year Age Groups

	All concussions			*MVC-related concussions*			*Sports-related concussions*		
Age group, years	*Odds ratio*	*95% Confidence interval*	p	*Odds ratio*	*95% Confidence interval*	p	*Odds ratio*	*95% Confidence interval*	p
0–4	1.49	1.33–1.67	0.00	1.66	1.19–2.33	0.00	1.26	1.01–1.58	0.04
5–9	1.39	1.28–1.51	0.00	1.52	1.18–1.95	0.00	1.19	1.02–1.38	0.03
10–14	1.30	1.23–1.38	0.00	1.39	1.17–1.66	0.00	1.12	1.02–1.23	0.02
15–19	1.23	1.19–1.27	0.00	1.29	1.15–1.45	0.00	1.07	1.01–1.13	0.03
20–24	1.16	1.14–1.19	0.00	1.21	1.12–1.3	0.00	1.03	0.98–1.08	0.33
25–29	1.11	1.08–1.13	0.00	1.14	1.08–1.2	0.00	0.99	0.94–1.06	0.84
30–34	1.06	1.03–1.09	0.00	1.08	1.03–1.14	0.00	0.97	0.91–1.04	0.41
35–39	1.02	0.99–1.05	0.17	1.04	0.98–1.1	0.25	0.96	0.89–1.03	0.23
40–44	0.99	0.96–1.02	0.47	1.00	0.94–1.07	0.99	0.95	0.88–1.02	0.18
45–49	0.96	0.93–0.99	0.01	0.97	0.91–1.04	0.43	0.95	0.88–1.03	0.23
50–54	0.94	0.91–0.97	0.00	0.96	0.9–1.02	0.17	0.96	0.87–1.06	0.44
55–59	0.93	0.89–0.96	0.00	0.95	0.88–1.02	0.14	0.98	0.85–1.13	0.76
60–64	0.92	0.87–0.96	0.00	0.95	0.86–1.04	0.26	1.00	0.82–1.22	0.97
65–69	0.91	0.85–0.98	0.01	0.95	0.83–1.1	0.50	1.04	0.79–1.36	0.78
70–74	0.91	0.82–1.01	0.06	0.97	0.79–1.19	0.74	1.08	0.76–1.55	0.66
75–79	0.91	0.8–1.04	0.19	0.99	0.75–1.31	0.94	1.14	0.72–1.8	0.57
80–84	0.92	0.78–1.1	0.36	1.02	0.71–1.47	0.91	1.21	0.68–2.14	0.51
85+	0.94	0.76–1.16	0.56	1.06	0.67–1.7	0.80	1.30	0.65–2.6	0.47
