# Supplementary material for: Neck Injury Comorbidity in Concussion-Related Emergency Department Visits: A Population-Based Study of Sex Differences Across the Life Span
Source: J Womens Health (Larchmt). 2019 Apr 22;28(4):473–82. doi: 10.1089/jwh.2018.7282 (PMC6482894; doi:10.1089/jwh.2018.7282)
Supplement: Supplemental data [file Supp_Table2.pdf]

Supplementary Table S2. Logistic Regression Model Examining the Odds of Comorbid Neck Injury for Sex (Reference = Male) Among Patients with a First Concussion-Related Emergency Department Visit in Ontario, Canada, 2002/2003–2011/012, by 5-Year Age Groups

	All concussions			*MVC-related concussions*			*Sports-related concussions*		
Age group, years	*Odds ratio*	*95% Confidence interval*	p	*Odds ratio*	*95% Confidence interval*	p	*Odds ratio*	*95% Confidence interval*	p
0–4	1.05	0.73–1.51	0.79	1.26	0.38–4.19	0.70	0.90	0.45–1.82	0.77
5–9	1.27	1.02–1.57	0.03	1.39	0.65–2.94	0.39	1.21	0.84–1.73	0.31
10–14	1.45	1.27–1.64	0.00	1.48	0.95–2.29	0.08	1.47	1.23–1.76	0.00
15–19	1.57	1.42–1.73	0.00	1.54	1.19–1.99	0.00	1.65	1.41–1.93	0.00
20–24	1.63	1.47–1.8	0.00	1.56	1.27–1.92	0.00	1.73	1.42–2.1	0.00
25–29	1.62	1.46–1.81	0.00	1.56	1.26–1.92	0.00	1.72	1.38–2.14	0.00
30–34	1.57	1.41–1.75	0.00	1.53	1.23–1.88	0.00	1.63	1.28–2.09	0.00
35–39	1.48	1.33–1.64	0.00	1.47	1.2–1.8	0.00	1.51	1.13–2.02	0.01
40–44	1.37	1.22–1.53	0.00	1.40	1.14–1.72	0.00	1.38	0.97–1.95	0.07
45–49	1.25	1.1–1.41	0.00	1.32	1.05–1.65	0.02	1.25	0.83–1.88	0.29
50–54	1.13	0.98–1.3	0.10	1.23	0.95–1.6	0.12	1.15	0.72–1.84	0.57
55–59	1.02	0.87–1.2	0.80	1.14	0.85–1.55	0.38	1.08	0.62–1.87	0.78
60–64	0.93	0.77–1.11	0.42	1.06	0.75–1.49	0.73	1.06	0.53–2.09	0.88
65–69	0.85	0.69–1.05	0.13	0.98	0.66–1.45	0.93	1.09	0.43–2.75	0.86
70–74	0.80	0.62–1.03	0.08	0.91	0.56–1.49	0.71	1.19	0.32–4.48	0.80
75–79	0.76	0.54–1.08	0.13	0.85	0.43–1.69	0.64	1.41	0.21–9.44	0.72
80–84	0.75	0.46–1.23	0.26	0.80	0.29–2.18	0.66	1.83	0.13–26.46	0.66
85+	0.77	0.38–1.55	0.47	0.76	0.17–3.28	0.71	2.63	0.07–101.64	0.60
