# Supplementary material for: Neck Injury Comorbidity in Concussion-Related Emergency Department Visits: A Population-Based Study of Sex Differences Across the Life Span
Source: J Womens Health (Larchmt). 2019 Apr 22;28(4):473–82. doi: 10.1089/jwh.2018.7282 (PMC6482894; doi:10.1089/jwh.2018.7282)
Supplement: Supplemental data [file Supp_Table3.pdf]

Supplementary Table S3. Logistic Regression Model Examining the Odds of Comorbid Neck Injury for Age Among Female Patients with a First Concussion-Related Emergency Department Visit in Ontario, Canada, 2002/2003–2011/2012, by 5-Year Age Groups

	All concussions			*MVC-related concussions*			*Sports-related concussions*		
Age group, years	*Odds ratio*	*95% Confidence interval*	p	*Odds ratio*	*95% Confidence interval*	p	*Odds ratio*	*95% Confidence interval*	p
0–4	1.80	1.6–2.03	0.00	1.82	1.31–2.54	0.00	1.69	1.26–2.27	0.00
5–9	1.59	1.46–1.73	0.00	1.62	1.27–2.07	0.00	1.44	1.19–1.76	0.00
10–14	1.41	1.33–1.5	0.00	1.45	1.22–1.72	0.00	1.26	1.11–1.42	0.00
15–19	1.27	1.23–1.32	0.00	1.31	1.17–1.47	0.00	1.12	1.04–1.21	0.00
20–24	1.16	1.13–1.19	0.00	1.20	1.12–1.29	0.00	1.02	0.96–1.08	0.57
25–29	1.07	1.05–1.1	0.00	1.11	1.06–1.17	0.00	0.95	0.88–1.02	0.14
30–34	1.00	0.98–1.03	1.00	1.04	0.99–1.09	0.09	0.90	0.83–0.98	0.01
35–39	0.94	0.92–0.97	0.00	0.99	0.93–1.04	0.62	0.87	0.8–0.95	0.00
40–44	0.90	0.87–0.93	0.00	0.94	0.89–1	0.07	0.86	0.79–0.95	0.00
45–49	0.87	0.85–0.9	0.00	0.91	0.86–0.97	0.00	0.87	0.79–0.97	0.01
50–54	0.85	0.83–0.88	0.00	0.89	0.84–0.95	0.00	0.90	0.78–1.05	0.18
55–59	0.84	0.81–0.87	0.00	0.88	0.82–0.94	0.00	0.96	0.77–1.19	0.68
60–64	0.84	0.8–0.88	0.00	0.88	0.8–0.96	0.01	1.03	0.76–1.4	0.84
65–69	0.85	0.8–0.91	0.00	0.88	0.77–1.02	0.08	1.14	0.75–1.73	0.54
70–74	0.87	0.8–0.96	0.00	0.90	0.74–1.1	0.31	1.28	0.74–2.22	0.37
75–79	0.90	0.8–1.02	0.10	0.93	0.71–1.22	0.59	1.48	0.74–2.96	0.27
80–84	0.95	0.81–1.11	0.48	0.97	0.68–1.39	0.85	1.74	0.74–4.12	0.21
85+	1.00	0.82–1.22	0.99	1.02	0.64–1.61	0.94	2.09	0.74–5.96	0.17
